# Modified quantitative and volumetric response evaluation criteria for patients with hepatocellular carcinoma after transarterial chemoembolization

**DOI:** 10.3389/fonc.2023.957722

**Published:** 2023-01-25

**Authors:** Jiachen Xu, Yu Yin, Jun Yang, Li Chen, Zhi Li, Jian Shen, Wansheng Wang, Caifang Ni

**Affiliations:** Department of Interventional Radiology, First Affiliated Hospital of Soochow University, Suzhou, China

**Keywords:** hepatocellular carcinoma, transarterial chemoembolization, tumor response, European Association for Study of the Liver, modified response evaluation criteria in solid tumors

## Abstract

**Objective:**

This study aimed to investigate the cutoff value of quantitative and volumetric response evaluation criteria for patients with hepatocellular carcinoma (HCC) after transarterial chemoembolization (TACE) and compare the performance of the modified criteria to one-dimensional criteria in survival prediction.

**Methods:**

A retrospective single-center study was performed for treatment-naive patients with HCC who underwent initial TACE between June 2015 and June 2019. Treatment response assessment was performed after the first observation by contrast CT or MRI, with the measurement of diameters by modified Response Evaluation Criteria in Solid Tumors (mRECIST) and volumes by quantitative European Association for Study of the Liver (qEASL). Overall survival (OS) was the primary endpoint of this study. The new cutoff value for volumetric response evaluation criteria was created using restricted cubic splines. The performance of modified qEASL (mqEASL, with the new cutoff value) and mRECIST on survival prediction was compared by Cox regression models in internal and external validation.

**Results:**

A total of 129 patients (mean age, 60 years ± 11 [standard deviation]; 111 men) were included and divided into training (n=90) and validation (n=39) cohorts. The cutoff value for the viable volume reduction was set at 57.0%. The mqEASL enabled separation of non-responders and responders in terms of median OS (p<0.001), 11.2 months (95% CI, 8.5–17.2 months) vs. 31.5 months (95% CI, 25.5–44.0 months). Two multivariate models were developed with independent prognostic factors (tumor response, metastasis, portal vein tumor thrombus, and subsequent treatment) to predict OS. Model 2 (for mqEASL) had a greater Harrel’s C index, higher time-dependent area under the receiving operator characteristic curve (AUROC), and more precise calibration on 6-month survival rates than Model 1 (for mRECIST).

**Conclusions:**

With the modified cutoff value, the quantitative and volumetric response of HCC patients to TACE becomes a precise predictor of overall survival. Further studies are needed to verify this modification before application in clinical practice.

## 1 Introduction

Hepatocellular carcinoma (HCC) was the sixth most commonly diagnosed cancer and the third leading cause of cancer death worldwide in 2020, with approximately 906,000 new cases and 830,000 deaths, according to statistics published by the World Health Organization (1). Most patients with HCC lost the opportunity to undergo curative treatments such as resection and liver transplantation because they had intermediate- or advanced-stage disease when diagnosed with HCC (2–4). Transarterial chemoembolization (TACE) is one of the most commonly recommended treatments for these patients according to clinical practice guidelines from various nations and regions (5–9). Furthermore, patients who showed a better response to TACE treatment in repeated sessions, as evaluated by posttreatment imaging, are likely to have more prolonged overall survival (10–12).

Among the response evaluation criteria, the modified Response Evaluation Criteria in Solid Tumors (mRECIST) is most commonly used in patients with HCC undergoing TACE (13, 14). Because chemoembolization often induces tumor necrosis rather than size shrinkage, a measurement of enhancing tumor size instead of the whole lesion has been shown to be more suitable for TACE. However, due to the nature of one-dimensional measurement, the sum of the diameters of enhancing tumors is just an approximate surrogate for the total viable tumor volume. To overcome the shortcomings of mRECIST, quantitative European Association for the Study of the Liver (qEASL) was proposed, which is a three-dimensional (3D) quantitative imaging analysis that was able to calculate viable tumor volume before and after treatment (15–17). The diagnostic accuracy of identifying tumor necrosis in HCC lesions was verified by a radiological–pathological correlation study (18). Moreover, several retrospective studies have validated the superiority of qEASL over other criteria in identifying responders and non-responders after not only TACE (19, 20) but also sorafenib (21) and Y90 radioembolization (22) in HCC patients.

However, the cutoff value for qEASL (65% of enhancing tumor volume reduction) in determining responders was derived from mRECIST (30% of maximum diameter reduction) and calculated using the formula V=4/3πr^3^ (19, 20). Few studies looked into a cutoff value for tumor volume change that was close to reality. As a result, we conducted a study to modify the qEASL cutoff value so that the response evaluation of HCC patients who underwent TACE could contribute more to survival prediction.

## 2 Materials and methods

### 2.1 Patient selection and data collection

**Figure 1:**
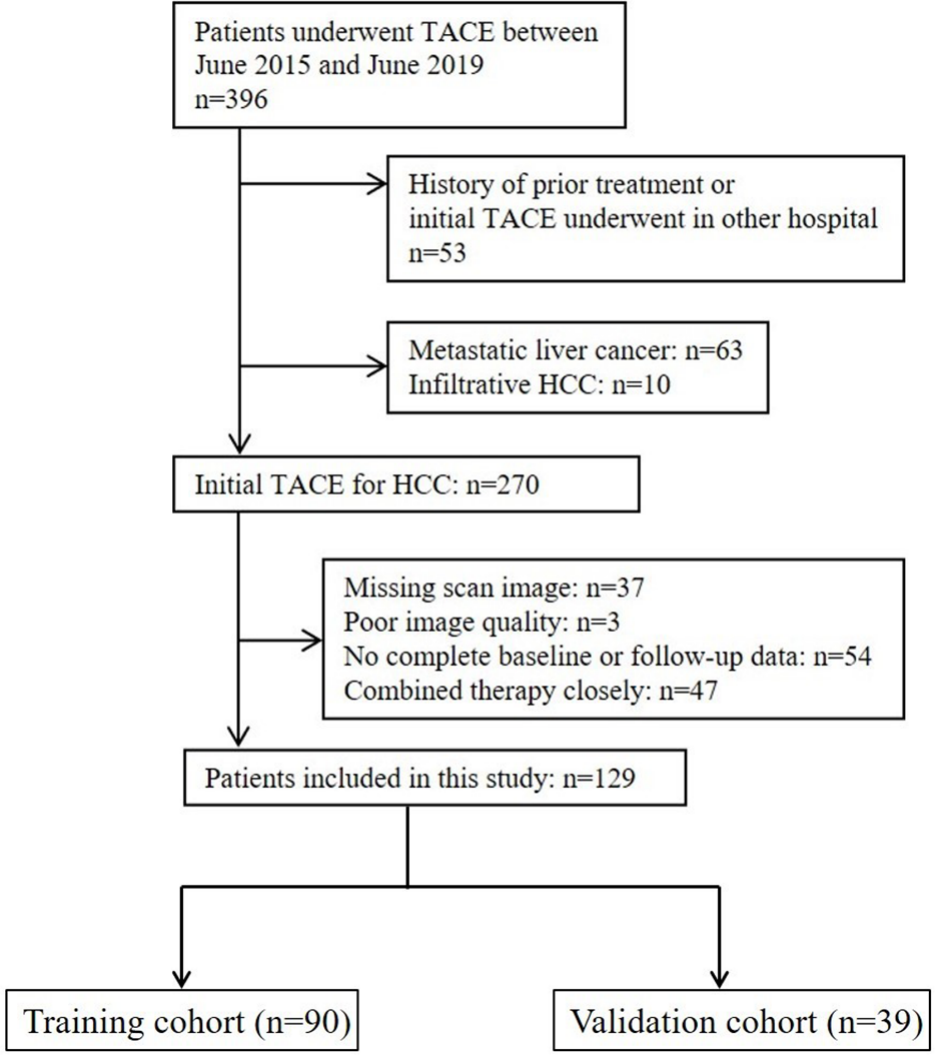
Flowchart of patients included and excluded in the study.

This retrospective study was approved by the Institutional Review Board, and the requirement for informed consent from patients was waived. The design of the study was in agreement with the Standards for Reporting of Diagnostic Accuracy guidelines. A list of 396 consecutive patients who underwent TACE at our institution between June 2015 and June 2019 was collected and checked for eligibility (**Figure 1**). The inclusion criteria were as follows: (a) age ≥18 years old, (b) HCC diagnosis (histological confirmation or clinical–radiological results of early enhancement followed by quick washout on dynamic liver imaging) in accordance with EASL or American Association for the Study of Liver Diseases guidelines (5, 7), (c) preserved liver function with Child–Pugh Class A or B, (d) Eastern Cooperative Oncology Group (ECOG) performance status (PS) ≤2, and (e) TACE chosen as the initial treatment. The exclusion criteria were as follows: (a) infiltrative HCC, (b) no complete pre- and posttreatment images or poor image quality with motion artifacts, (c) no baseline and/or follow-up data, and (d) a history of prior treatment other than TACE. The endpoint of this study was overall survival, and follow-up was terminated on 1 June 2021. Enrolled patients were randomly assigned to either the training or the validation cohorts at a ratio of 7:3.

### 2.2 Treatment

All TACE procedures were performed by three interventional radiologists (Z.L., J.S., and W.W., with TACE experience for 10, 15, and 20 years, respectively), following technical recommendations (23). Briefly, a 2.7-Fr microcatheter (Progreat, Terumo, Japan) was advanced, and the tip of the catheter was superselectively placed in the subsegmental tumor-feeding vessel (s). For conventional TACE, a water-in-oil emulsion with two volumes of lipiodol (up to 15 ml, Lipiodol Ultrafluid, Guerbet, France) and one volume of doxorubicin (50 mg/m^2^ surface area) was infused, followed by embolization with 100–300 μm gelatin sponge particles (Ailicon Pharmaceutical Technology Co. Ltd., Hangzhou, China). For TACE with drug-eluting beads, a total of 80 mg of doxorubicin at a concentration of 20 mg/ml was loaded into a vial of 100–300 μm CalliSphere beads (Jiangsu Hengrui Medicine Co., Ltd., China) and mixed with 10 ml of nonionic contrast (Iodixanol, Jiangsu Hengrui Medicine Co., Ltd., China). Embolization was not stopped until the stasis of blood flow in the target artery was obtained. TACE treatment was repeated on demand every 6–8 weeks when sequential images showed evident enhancing lesions and was terminated when an objective response was not reached after consecutive sessions. A multidisciplinary liver tumor board determined subsequent treatments (including resection, radiofrequency ablation, internal radiotherapy, targeted therapy, and immunotherapy) based on changes in the patients’ condition.

### 2.3 Image acquisition

Patients underwent either multiphasic computed tomography (CT) or magnetic resonance (MR) scans at baseline (1–2 weeks before initial TACE treatment) and follow-up. Assessment scans were performed 6–8 weeks after initial TACE. Multiphasic contrast-enhanced images on CT were obtained using multidetector CT scanners (Siemens Medical Solutions, Germany; Philips Healthcare, The Netherlands). MR imaging was performed using 3.0-Tesla MR systems (Siemens, Erlangen, Germany, parameters: TR/TE, 3.3/1.16; a 13° flip angle; matrix, 256×192; slice thickness, 2.5 mm). Multiphasic enhanced images, including arterial phase, portal venous phase, and delayed images, were obtained 20, 70, and 180 s after all intravenous contrast (iodixanol for CT and gadodiamide for MR) was administered.

### 2.4 Tumor response assessment

Two interventional radiologists (J.Y., with 3 years of experience, and Y.Y., with 5 years of experience) who were blinded to the patients’ medical history and outcomes independently and retrospectively reviewed the scan images. Intrahepatic target tumors were identified if their longest diameter ≥1 cm, with typical intratumoral arterial enhancement, and received standardized embolization treatment. The tumor response after the first TACE was used as a prognostic factor in this study. For mRECIST, the sum of the largest diameters of target-enhancing tumors (D), avoiding major areas of internal necrosis, was measured at baseline (BL) and follow-up (UP). The percentage of diameter change was calculated as $DC=\frac{D\;\left(UP\right)-D\;\left(BL\right)}{D\;\left(BL\right)}\times \;100\left[\%\right]$ . Patients were stratified into complete response (CR, the complete disappearance of all target tumor enhancement), partial response (PR, at least a 30% decrease in the sum of the largest viable tumor diameters), progressive disease (PD, at least a 20% increase in the sum of the largest viable tumor diameters, or new intrahepatic lesions), and stable disease (SD, neither PR nor PD). Responders included patients with CR and PR, while patients with SD and PD were divided into non-responders.

**Figure 2:**
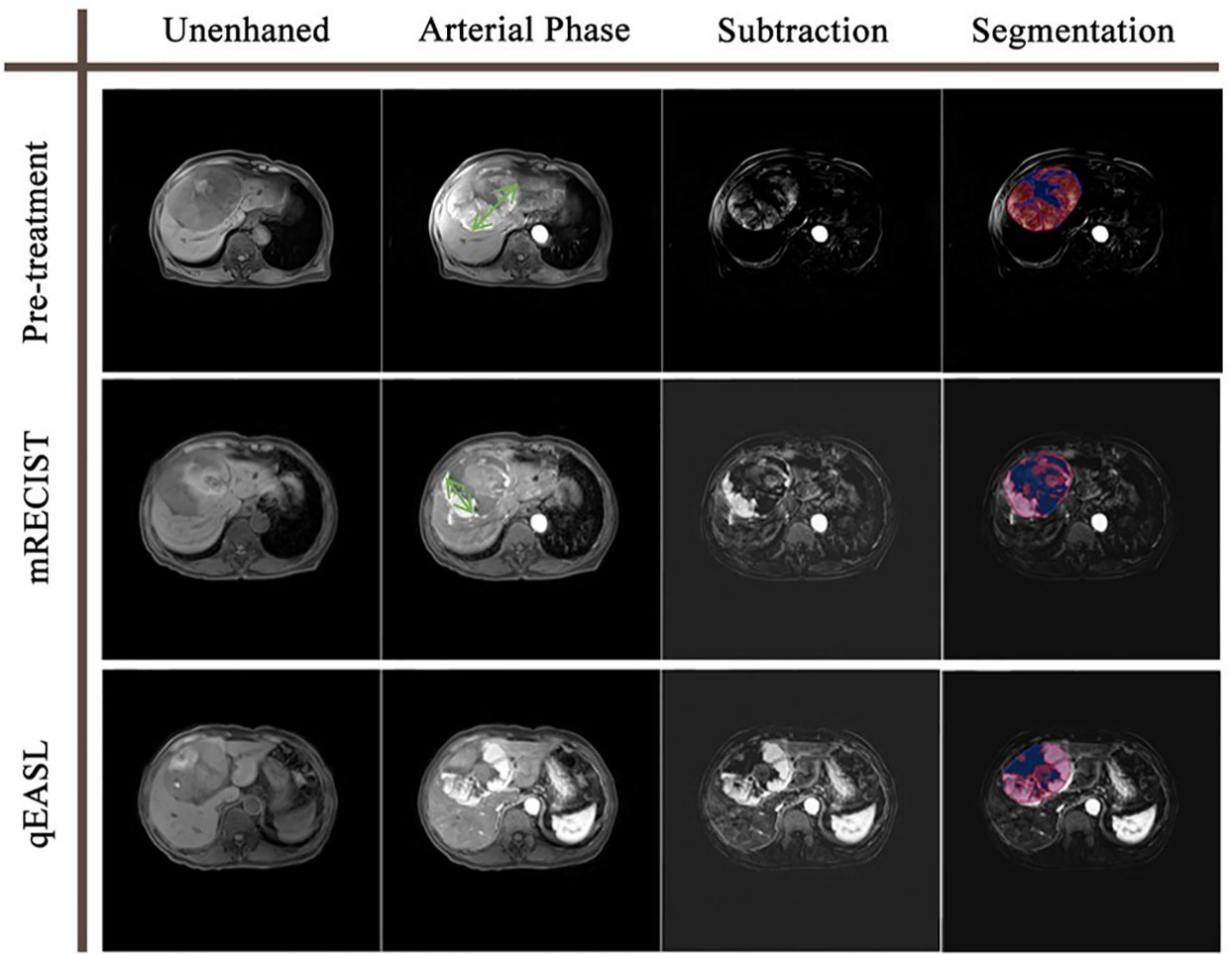
Tumor response evaluation based on the contrast-enhanced MR of a 78-year-old man with one HCC tumor. The green double-sided arrows represent the measurement of the diameter of the largest viable tumor on arterial phase images. The color maps represent the segmentation using the qEASL method, where blue maps illustrate inactive areas and red maps indicate viable tumors. The viable tumor volume was 494.2 cm^3^ at baseline and 292.5 cm^3^ after the initial TACE. The 40.8% viable tumor volume reduction suggested that this patient was a non-responder to TACE. In comparison, the diameter of the viable tumor was 11.5 cm at baseline and 6.7 cm after the initial TACE. The 41.7% diameter reduction suggested that this patient was a responder to TACE. The disagreement of the two evaluation methods may result from the irregular shape of the necrosis zone.

Quantitative EASL was performed using 3D Slicer software (https://www.slicer.org), a free-to-use platform for quantitative imaging analysis (24), following the principles described previously (15–17). Briefly, semiautomatic 3D tumor segmentation (Seg1) was performed on arterial phase enhanced images. After subtracting unenhanced images from enhanced images to remove background value, the enhancement value of liver parenchyma (as the threshold) was calculated by averaging values of three points of surrounding healthy tissues selected by experienced radiologists. A threshold tool was used to automatically segment voxels within Seg1 where the enhancement values were greater than the threshold. The volume of new segmentation (Seg2) was calculated to represent the viable tumor volume (VTV). Volume-based qEASL was adopted in this study (**Figure 2**). Both VTV at baseline (BL) and follow-up (UP) were collected to calculate the percentage of viable tumor volume change (VC): $\text{VC}=\frac{VTV\;\left(UP\right)-VTV\;\left(BL\right)}{VTV\;\left(BL\right)}\times \;100\;\left[\%\right]$ . Patients were divided into responders and non-responders with a cutoff value created in restricted cubic spline analysis as described below. Patients with new lesions were identified as non-responders (with progressive disease) and were excluded from the exploration of the cutoff value. Briefly, when a reduction in viable tumor volume reached or was greater than the cutoff value, the patient was classified as responder in mqEASL. On the contrary, when the criteria of responder was not reached or new lesions occurred, the patient was classified as non-responder in mqEASL.

### 2.5 Statistical analysis

Continuous variables are presented as the means with standard deviations or medians with interquartile ranges (IQRs) and were compared by Student’s t-test or the Mann–Whitney U-test. Categorical variables were summarized as numbers with percentages and compared by Fisher’s exact test. Correlations between response evaluation indexes were presented in scatter plots and fitted using a linear regression model. The association between volume change and hazard ratio of death was flexibly modeled by using four-knot restricted cubic splines. The volume change value whose corresponding hazard ratio of death equaled 1 was selected as the cutoff value to stratify responders and non-responders in modified qEASL (mqEASL). The evaluation agreement between mRECIST and mqEASL was assessed by the McNemar test, and the kappa statistic was calculated. Poor, moderate, and excellent agreement was judged by kappa values of <0.4, 0.4–0.75, and >0.75, respectively (25).

Overall survival (OS) was calculated from the day of the first TACE session to the date of death from any cause. Patients were censored at the last follow-up time point or the end of the observation period if they were lost to follow-up or still alive. Survival curves were estimated with the Kaplan–Meier method and compared with the log-rank test. The Cox proportional hazards model was used to identify predictors that have a significant influence on the survival of patients in both univariate and multivariate analyses. To compare the predictive performance of mRECIST and mqEASL in terms of overall survival, two Cox regression models were built based on the training cohort with pretreatment predictors together with posttreatment response markers evaluated by either mRECIST (Model 1) or mqEASL (Model 2). The discrimination and calibration of the two models were measured and compared in both the training and validation cohorts using Harrel’s C index, area under the time-dependent receiving operator characteristic curve (AUROC), and calibration curves. All statistical tests were conducted at the two-sided 5% significance level using R version 4.1.0.

## 3 Results

### 3.1 Patient characteristics

A group of 129 patients were included and divided into training (n=90) and validation (n=39) cohorts (**Figure 1**). The characteristics of the patients are summarized and compared in **Table 1**. The majority of HCC patients were male (training: n=79 [87.8%], validation: n=32 [82.1%]). Most patients tested positive for hepatitis virus B infection (training: n=75 [83.3%], validation: n=26 [66.7%]). Over two-thirds of the patients had stage A or B disease, according to the Barcelona Clinic for Liver Cancer (BCLC) system (training: n=63 [70.0%], validation: n=27 [69.2%]). Characteristics except etiology (p=0.044) were comparable between the two cohorts.

**Table 1:** Baseline patient characteristics.

Patient Characteristic	Training	Validation	p-value
**(N=129)**	n=90	n=39	
**Sex**			0.414
Male/female	79/11 (87.8%/12.2%)	32/7 (82.1%/17.9%)	
**Age** (year, mean ± SD)	59 ± 11	63 ± 10	0.079
**Etiology**			0.044
None/HBV/other	8/75/7 (8.9%/83.3%/7.8%)	10/26/3 (25.6%/66.7%/7.7%)	
**ECOG PS**			0.658
0/1/2	73/15/2 (81.1%/16.7%/2.2%)	30/7/2 (76.9%/17.9%/5.1%)	
**Child–Pugh**			1.000
A/B	81/9 (90.0%/10.0%)	36/3 (92.3%/7.7%)	
**ALBI**			1.000
1/2/3	36/51/3 (40.0%/56.7%/3.3%)	16/22/1 (41.0%/56.4%/2.6%)	
**Tumor number** (median, IQR)	2 (1~3)	2 (1~3)	0.502
**Diameter of largest tumor**			1.000
<5 cm/≥5 cm	49/41 (54.4%/45.6%)	21/18 (53.8%/46.2%)	
**Up-to-seven**			0.702
In/Beyond	49/41 (54.4%/45.6%)	23/16 (59%/41%)	
**AFP**			0.563
<200 ng/ml**/**≥200 ng/ml	54/36 (60.0%/40.0%)	21/18 (53.8%/46.2%)	
**Metastasis**			0.635
Negative/positive	73/17 (81.1%/18.9%)	30/9 (76.9%/23.1%)	
**PVTT**			1.000
Negative/positive	74/16 (82.2%/17.8%)	32/7 (82.1%/17.9%)	
**BCLC stage**			0.233
A/B/C	25/38/27 (27.8%/42.2%/30.0%)	16/11/12 (41.0%/28.2%/30.8%)	
**TACE type**			0.806
Conventional/DEB	74/16 (82.2%/17.8%)	31/8 (79.5%/20.5%)	
**TACE sessions**(median, IQR)	3 (2~4)	3 (2~4)	0.733
**Image interval** (months, median, IQR)	2 (1.5~2.5)	2 (1.5~2.5)	0.633
**mRECIST**			0.548
Complete response	14 (15.6%)	9 (23.1%)	
Partial response	34 (37.8%)	11 (28.2%)	
Stable disease	27 (30.0%)	14 (35.9%)	
Progressive disease	15 (16.7%)	5 (12.8%)	
**Subsequent treatment**			0.194
None	47 (52.2%)	20 (51.3%)	
Locoregional	30 (33.3%)	8 (20.5%)	
Systemic	7 (7.8%)	5 (12.8%)	
Combined	6 (6.7%)	6 (15.4%)	

Unless otherwise indicated, data are numbers of patients, and data in parentheses are percentages. HBV, hepatitis B virus infection; ECOG, Eastern Cooperative Oncology Group; PS, performance status; ALBI, albumin–bilirubin scores; IQR, interquartile range; AFP, alpha fetoprotein; PVTT, portal vein tumor thrombi; BCLC, Barcelona Clinic Liver Cancer; DEB, drug eluting beads; mRECIST, modified Response Evaluation Criteria in Solid Tumors.

### 3.2 Cutoff value of viable tumor volume change

**Figure 3:**
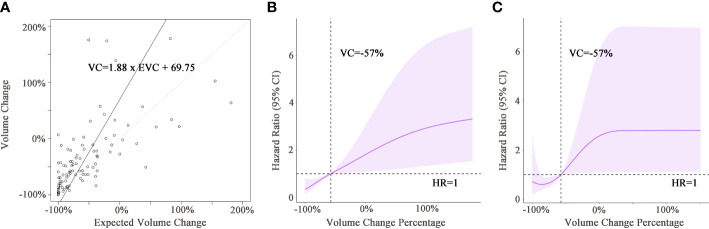
**(A)** The scatter plot shows the correlation between the expected viable volume change and actual viable volume change. The full line was fitted by linear regression models, with the equation presented in the rectangular frame. The dotted line is the line of reference for VC = EVC. **(B, C)** Graphs of restricted cubic spline with 95% confidence intervals for volume change and hazard ratio of death in the training cohort **(B)** and validation cohort **(C)**. Black dotted lines indicate that the hazard ratio of death equaled 1 when the volume change was −57% in both cohorts. VC, viable tumor volume change; EVC, expected viable tumor volume change; HR, hazard ratio.

The expected volume change (EVC) was calculated by the following formula: EVC=[(1+Diameter change)^3−1]×100(%). The relationships between EVC and VC are depicted in the scatter plot (**Figure 3A**). The fitted linear regression equation for EVC and VC was VC=1.88×EVC+69.75 (far from the ideal: VC=EVC). Simply using the expected volume change as a substitute for the actual viable volume change would cause imprecision. The relationship between viable tumor volume change and risk of mortality in the training and validation cohorts is shown in **Figures 3B, C**. A decrease in viable volume of more than 57.0% was revealed to be a protective factor against mortality. On the other hand, a decrease in viable volume that did not reach 57.0% or an increase in viable volume suggested a quick increase in the risk of death. Consequently, a decrease of 57.0% was selected as the cutoff value in mqEASL (responder, ≥57.0% decrease; non-responder, responder criteria not met or new lesion). The evaluation agreement of mRECIST and mqEASL in all enrolled patients is summarized in **Supplementary Table S1**. The kappa value was 0.5977 (95% CI, 0.4596–0.7359), indicating only moderate agreement (McNemar test p=0.327).

### 3.3 Survival analysis

**Figure 4:**
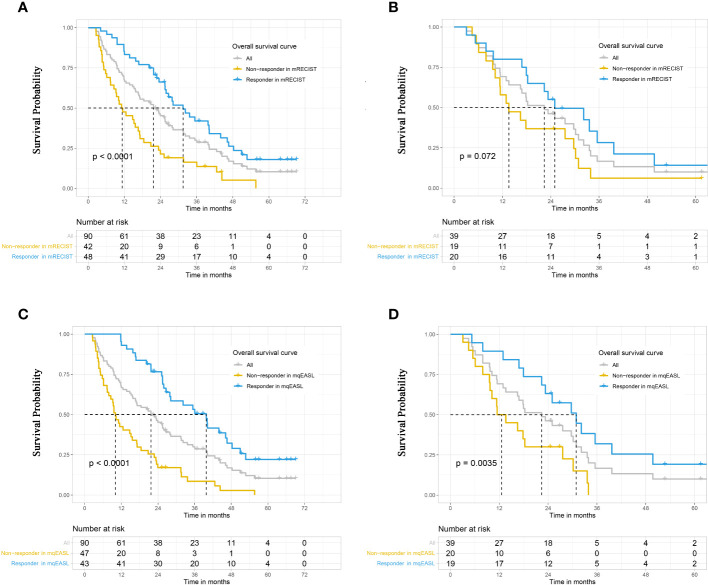
Kaplan–Meier curves to compare survival between different groups in the training cohort **(A, B)** and validation cohort **(C, D)**. Responders and non-responders were stratified after initial TACE according to tumor response, which was evaluated by the methods of mRECIST **(A, C)** and mqEASL **(B, D)**. The result of the log-rank test (p-value) is marked on each graph. mRECIST, modified Response Evaluation Criteria in Solid Tumors; mqEASL, modified quantitative European Association for Study of the Liver.

During the observation period, 106 patients died (training: n=74, validation: n=32), and 23 patients were censored due to the termination of the observation period (training: n=16, validation: n=7). The median OS of the entire group was 22.4 months (95% CI, 17.0–26.6 months). Notably, in the training cohort (**Figures 4A, B**), the mqEASL enabled stronger separation of non-responders and responders in terms of median OS, 11.2 months (95% CI, 8.5–17.2 months) vs. 31.5 months (95% CI, 25.5–44.0 months) for mRECIST (p<0.001) and 10.1 months (95% CI, 7.9–17.0 months) vs. 39.8 months (95% CI, 27.9–48.3 months) for mqEASL (p<0.001). In the validation cohort (**Figures 4C, D**), the difference in overall survival between non-responders and responders in mRECIST was not significant, 13.6 months (95% CI, 11.4–30.2 months) vs. 25.0 months (95% CI, 18.3–49.7 months) for mRECIST (p=0.072), and 12.5 months (95% CI, 9.7–30.2 months) vs. 30.9 months (95% CI, 23.3–NA months) for mqEASL (p=0.004).

### 3.4 Univariate and multivariate analyses

The results of univariate and multivariate analyses in the training cohort are summarized in **Supplementary Table S2**. Independent prognostic factors (not considering response markers) were identified as follows: presence of metastasis (p<0.001), presence of portal vein tumor thrombus (p=0.002), and subsequent treatment (p=0.034).

### 3.5 Comparison of mRECIST and mqEASL in survival prediction

To compare the survival prediction performance of mRECIST and mqEASL in a multivariate setting, two Cox regression models (**Supplementary Table S3**) were created as follows:

Model 1: linear predictor (LP) =1.68×Metastasis + 1.43×PVTT − 0.47×Subsequent treatment − 1.08×Responder 1

Model 2: LP=1.88×Metastasis + 0.92×PVTT − 0.36×Subsequent treatment − 1.41×Responder 2

“Metastasis,” “PVTT,” and “responder” are binary variables that have a value of 0 for no metastasis, no PVTT, and non-responder and a value of 1 for metastasis, PVTT, and responder. “Subsequent treatment” is an ordinal categorical variable with a value of 0 for none, 1 for locoregional therapy, 2 for systemic therapy, and 3 for combined therapy.

**Figure 5:**
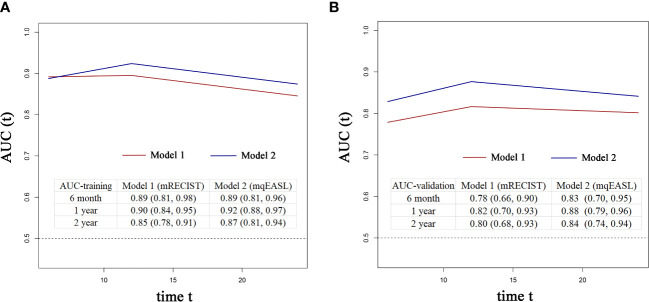
Time-dependent AUROC values of Model 1 and Model 2 in the training cohort **(A)** and validation cohort **(B)**. The inserted tables show the AUROC values with 95% confidence intervals of the models at different time points. AUC, area under the curve.

**Figure 6:**
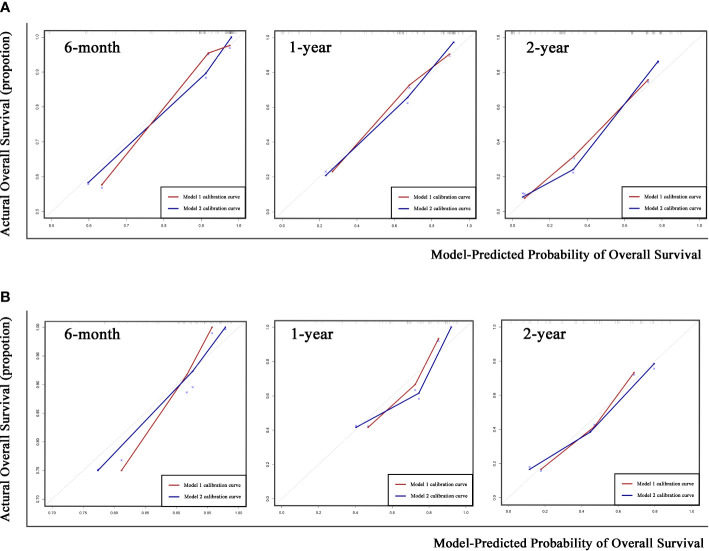
Calibration curves of the two prediction models for 6-month, 1-year, and 2-year overall survival (OS) in the training cohort **(A)** and validation cohort **(B)**. Gray dotted lines represent the calibration curve of the most ideal predictive method.

The coefficient of Responder 2 (based on mqEASL) was greater than that of Responder 1 (based on mRECIST). The Harrel’s C index of Model 2 was higher than that of Model 1 in both the training and validation cohorts (training [1 vs 2]: 0.778 ± 0.026 vs. 0.795 ± 0.024, validation [1 vs 2]: 0.725 ± 0.043 vs. 0.759 ± 0.041). The 6-month, 1-year, and 2-year AUROC values of Model 2 were also higher than those of Model 1 in both the training (**Figure 5A**) and validation (**Figure 5B**) cohorts, suggesting favorable discrimination of mqEASL over mRECIST. The calibration curves of the two models are shown in **Figure 6**. Model 2 showed better consistency between the predicted probability of 6-month and 1-year OS and the actual outcomes in the training cohort (**Figure 6A**). The consistency of Model 2 in 6-month OS was further confirmed by external validation (**Figure 6B**).

## 4 Discussion

Our study proposed a valid cutoff value for quantitative and volumetric tumor response evaluation criteria. The modified qEASL criteria are competent for identifying non-responders to TACE and predicting overall survival.

To date, a variety of therapies have been available for patients with HCC at different disease stages. The prompt transition from ineffective therapy to other attempts is essential for patients’ overall survival. Transarterial chemoembolization is one of the most effective and widely used locoregional therapies. Several scoring systems were developed to identify patients who were unlikely to benefit from repeated TACE, such as the ART (Assessment for Retreatment with TACE) score and ABCR (Alpha fetal Protein, BCLC, Child–Pugh, Response) score (26–28). Within these scores, the radiological tumor response is a significant factor (29). When non-responders to TACE are identified early, systemic or combined therapies can be used before liver function deteriorates (30–33).

Currently used criteria for tumor response evaluation, including mRECIST and EASL, adopt 1- or 2-dimensional measurements to reflect tumor extent. In recent years, the newly developed qEASL criteria have demonstrated superiority in quantifying enhancing tumor volume (17, 18, 20–22). The example in **Figure 2** shows a familiar situation in clinical practice. The irregular shape of the internal necrosis area hindered the measurement of viable tumor diameter after TACE treatment, which could be overcome by a 3D measurement of viable tumor volume. In the ideal scenario, where tumor lesions are regular spheres and shrink symmetrically, a ≥30% decrease in diameter or a ≥50% decrease in section area approximately equaled a ≥65% decrease in volume. However, the actuality fell far short of the ideal. All of the former studies investigating qEASL adopted the calculated cutoff value (65% decrease in tumor volume) in dividing non-responders and responders after different treatments. Our study investigated the relationship between expected volume change and actual volume change (*VC*=1.88×*EVC*+69.75 ). The findings revealed that the calculated volume change of inhomogeneous liver tumors based on diameter change does not match reality. As a result, the calculated cutoff value for qEASL would cause response evaluation inaccuracy.

A new cutoff value for volumetric response evaluation (≥57.0% decrease in enhancing tumor volume for responders) was proposed in this study, with the aim that stratification of responders and non-responders could be more capable of predicting survival. The previously used cutoff value (65% decrease, qEASL) was stricter than the modified cutoff value (57.0% decrease, mqEASL). Under the stricter rules, some patients who could benefit from TACE would be classified as non-responders. It may cause increased sensitivity but decreased specificity. Furthermore, there was only moderate agreement between mRECIST and mqEASL (kappa value=0.5977), emphasizing the necessity of comparing the two criteria.

The performance of the mqEASL was confirmed in a multivariate way by both internal and external validations. The prediction model created with independent prognostic factors (metastasis, PVTT, and subsequent treatment) and the mqEASL response marker demonstrated superior discrimination and calibration than that with the mRECIST response marker. There was no significant difference in the calibration of the two models with regard to predicting the 2-year survival rate. This could be explained by the fact that the tumor response to the initial TACE was related to a better prognosis in the short term (6 months and 1 year). In contrast, the long-term survival outcomes were influenced by a variety of factors (34, 35). Different treatment modalities strongly affected the long-term survival. When HCC advanced into a systemic disease, systemic and combined therapies as recommended can actually prolonged patients` overall survival. One doubt about the mqEASL is that the postprocessing steps take a lot of time. In our experience, once the segment of whole tumor lesion(s) and the threshold were confirmed, the volume of viable tumor was automatically calculated by the software. It takes approximately 5–20 min for each patient. The accurate evaluation of tumor burden instead of using diameter for substitution requires more time. The development of artificial intelligence will make mqEASL easier to perform in clinical practice.

There are some limitations to our study. First, the nature of retrospective studies introduced unavoidable biases. Second, the sample size was barely enough. The total number of deaths (74/90) limited the number of candidate variables that could be used in the multivariate Cox regression analysis to seven, with a ratio of 10 events per variable. Moreover, a larger external validation cohort will be required before mqEASL can be applied in clinical practice. Third, since CT and MRI were parallelly adopted in clinical practice, we included both image types for evaluation. Previous studies have proven that the qEASL criteria can be applied to multiphasic CT or MRI (19, 20). However, a small portion of patients in our study received different types of scans at baseline and follow-up. Whether volumetric evaluation criteria could be applied in this situation needs more investigation. Lastly, two different type of TACE methods were applied to patients with HCC, which may increase the heterogeneity of this study. In further analysis, the proportion of responders between conventional and DEB TACE groups showed no significance (**Supplementary Table S4**). In the univariate analysis of prognostic factors for survival, TACE type failed to stand out (**Supplementary Table S2**). Thus, we supposed that the different TACE methods had little influence on tumor response and overall survival.

In conclusion, the modified volumetric and quantitative response evaluation criteria could enable more accurate identification of non-responders among HCC patients to TACE treatment. The new response marker was more competent to predict overall survival than mRECIST.

## Data availability statement

The original contributions presented in the study are included in the article/**Supplementary Material**. Further inquiries can be directed to the corresponding author.

## Ethics statement

Written informed consent was obtained from the individual(s) for the publication of any potentially identifiable images or data included in this article.

## Author contributions

Guarantor of integrity of entire study, CN. Study concepts/study design or data acquisition or data analysis/interpretation, all authors. Manuscript drafting or manuscript revision for important intellectual content, all authors. All authors agrees to ensure any questions related to the work are appropriately resolved. Literature research, JX, YY, JY, and LC. Clinical studies, YY, JY, ZL, JS, WW, and CN. Statistical analysis, JX, LC, and CN. Manuscript editing, JX, YY, JY, LC, and CN.
