# Supplementary material for: Modified quantitative and volumetric response evaluation criteria for patients with hepatocellular carcinoma after transarterial chemoembolization
Source: Front Oncol. 2023 Jan 25;13:957722. doi: 10.3389/fonc.2023.957722 (PMC9905806; doi:10.3389/fonc.2023.957722)
Supplement: Supplementary file 1 [file Table_1.docx]

Supplementary materials

Table 1 Agreement Analyses between mRECIST and mqEASL

|  |  | mRECIST | |
| --- | --- | --- | --- |
|  |  | Non-responder | Responder |
| mqEASL | Non-responder | 51 | 16 |
|  | Responder | 10 | 52 |
|  |  |  |  |
| Kappa index | 0.5977 | (0.4596, 0.7359) | Moderate agreement |
| McNemar P value | 0.3268 |  |  |

Note. —Cross table summarized the number of patients in different groups. The results of the tests for the cross table were listed. Data in parentheses are the 95% confidence intervals of the kappa index. mRECIST = modified Response Evaluation Criteria in Solid Tumors, mqEASL = modified quantitative European Association for Study of the Liver.

Table 2 Univariate and Multivariate Analysis of Prognostic Factors (training cohort)

| **Candidate Variables** | **Univariate (P value)** | **Multivariate (P value)** |
| --- | --- | --- |
| Gender | 0.231 |  |
| Age | 0.343 |  |
| Etiology | 0.799 |  |
| ECOG PS | ＜0.001† | 0.893 |
| Child-Pugh | 0.707 |  |
| ALBI | 0.66 |  |
| Tumor number | 0.946 |  |
| Diameter of largest tumor≥5cm | 0.026† | 0.807 |
| Up-to-seven | 0.251 |  |
| AFP≥200 | ＜0.001† | 0.214 |
| Metastasis | ＜0.001† | ＜0.001‡ |
| PVTT | ＜0.001† | 0.002‡ |
| BCLC stage | ＜0.001† | 0.809 |
| TACE type | 0.13 |  |
| Subsequent treatment | 0.008† | 0.034‡ |

Note. —Data are the Cox regression analysis P value. ECOG = Eastern Cooperative Oncology Group, PS = performance status, ALBI = Albumin-Bilirubin scores, AFP = alpha fetoprotein, PVTT = portal vein tumor thrombi, BCLC = Barcelona Clinic Liver Cancer.

† Variables with a P value of <0.10 in univariate analysis were enrolled in the multivariate analysis.

‡ Variables with a P value of <0.05 in multivariate analysis were identified as independent prognostic factors for overall survival.

Table 3 Development of Prognostic Models

|  | Coefficients | HR (95% CI) | P value |
| --- | --- | --- | --- |
| Metastasis | 1.68 | 5.39 (2.80, 10.36) | <0.001 |
| PVTT | 1.43 | 4.17 (2.12, 8.21) | <0.001 |
| Subsequent treatment | -0.47 | 0.63 (0.47, 0.83) | 0.001 |
| Responder 1 (mRECIST) | -1.08 | 0.34 (0.21, 0.57) | <0.001 |
| †LP1=1.68xMetastasis + 1.43xPVTT - 0.47xSubsequent treatment - 1.08xResponder 1 | | | |
|  |  |  |  |
| Metastasis | 1.88 | 6.57 (3.40, 12.70) | <0.001 |
| PVTT | 0.92 | 2.50 (1.30, 4.83) | 0.006 |
| Subsequent treatment | -0.36 | 0.70 (0.53, 0.92) | 0.009 |
| Responder 2 (qEASL) | -1.41 | 0.24 (0.14, 0.42) | <0.001 |
| †LP2=1.88xMetastasis + 0.92xPVTT - 0.36xSubsequent treatment - 1.41xResponder 2 | | | |

Note. —The coefficients of variables, hazard ratio with the 95% confidence intervals in parentheses, and P values estimated by Cox regression models are given. PVTT = portal vein tumor thrombi, mRECIST = modified Response Evaluation Criteria in Solid Tumors, mqEASL = modified quantitative European Association for Study of the Liver. LP = linear predictors.

† Prognostic models were developed based on the independent factors and their coefficients.

Table 4 Relationship between TACE methods and tumor response

|  | Conventional | DEB | P value |
| --- | --- | --- | --- |
| mRECIST |  |  |  |
| non-responder | 48 | 13 | 0.454 |
| responder | 57 | 11 |  |
| mqEASL |  |  |  |
| non-responder | 55 | 12 | 0.833 |
| responder | 50 | 12 |  |
